# Socioeconomic Status and Use of Outpatient Medical Care: The Case of Germany

**DOI:** 10.1371/journal.pone.0155982

**Published:** 2016-05-27

**Authors:** Jens Hoebel, Petra Rattay, Franziska Prütz, Alexander Rommel, Thomas Lampert

**Affiliations:** 1 Unit of Social Determinants of Health, Department of Epidemiology and Health Monitoring, Robert Koch Institute, Berlin, Germany; 2 Unit of Health Reporting, Department of Epidemiology and Health Monitoring, Robert Koch Institute, Berlin, Germany; National Institute of Health, ITALY

## Abstract

**Background:**

Socially disadvantaged people have an increased need for medical care due to a higher burden of health problems and chronic diseases. In Germany, outpatient care is chiefly provided by office-based general practitioners and specialists in private practice. People are free to choose the physician they prefer. In this study, national data were used to examine differences in the use of outpatient medical care by socioeconomic status (SES).

**Methods:**

The analyses were based on data from 6,754 participants in the Robert Koch Institute’s German Health Interview and Examination Survey for Adults (DEGS1) aged between 18 and 69 years. The number of outpatient physician visits during the past twelve months was assessed for several medical specializations. SES was determined based on education, occupation, and income. Associations between SES and physician visits were analysed using logistic regression and zero-truncated negative binomial regression for count data.

**Results:**

After adjusting for sociodemographic factors and health indicators, outpatients with low SES had more contacts with general practitioners than outpatients with high SES (men: incidence rate ratio [IRR] = 1.25; 95% confidence interval [CI] = 1.08–1.46; women: IRR = 1.20; 95% CI = 1.07–1.34). The use of specialists was lower in people with low SES than in those with high SES when sociodemographic factors and health indicators were adjusted for (men: odds ratio [OR] = 0.68; 95% CI = 0.51–0.91; women: OR = 0.56; 95% CI = 0.41–0.77). This applied particularly to specialists in internal medicine, dermatology, and gynaecology. The associations remained after additional adjustment for the type of health insurance and the regional density of office-based physicians.

**Conclusion:**

The findings suggest that socially disadvantaged people are seen by general practitioners more often than the socially better-off, who are more likely to visit a medical specialist. These differences may be due to differences in patient preferences, physician factors, physician-patient interaction, and potential barriers to accessing specialist care.

## Introduction

The German healthcare system differs from systems in other countries in many respects, especially in the outpatient care sector. Outpatient medical care in Germany is mainly delivered by office-based general practitioners (GPs) and specialists working in private practice, i.e. they are not employed by a hospital or medical centre, as is customary in many other countries. The fact that medical specialist care in Germany is provided both by office-based specialists and by specialists employed by hospitals can be regarded as a peculiarity of the German healthcare system. In other European countries, like the Netherlands, the UK or Spain, specialist care is predominantly provided in hospitals [1–3].

In Germany, the first point of contact in the professional healthcare system sought by people with health complaints is usually an outpatient physician's practice. People who are covered by statutory health insurance (about 85% of the population in Germany) are free to choose between all office-based physicians who have been accredited for the outpatient care of people with statutory health insurance [4, 5]. The German healthcare system is traditionally not a gatekeeping system. Therefore, people can consult office-based specialists of their own accord without a prior referral from a general practitioner [5]. Today, people with statutory health insurance in Germany have free-of-charge access to office-based physicians. Between 2004 and 2012, however, a €10 fee was charged for a patient's first visit to an outpatient physician's practice in every three-month period, payable personally by the person with statutory health insurance. Although this quarterly practice fee has since been abolished, people with statutory health insurance still have to make private co-payments for prescribed medicines and medical aids.

The need for medical services varies widely between different population groups. Many national and international studies agree that people with low socioeconomic status (SES) are affected by chronic diseases, physical complaints and mental health problems more frequently than people with higher SES [6–9]. As a result, they also have a greater need for the services provided by the medical care system and use them more frequently [10]. While socioeconomic differences in health and morbidity have by now been extensively studied, the question of social determinants in healthcare has in Germany only become a focus of public-health research in the last few years [11, 12]. With respect to outpatient healthcare in general, a systematic review indicates that people with lower SES use GP services more frequently and specialist medical services more rarely than people with higher SES [12]. In particular, it is reported that people with high SES are more likely to use medical prevention services, such as examinations for the early detection of diseases, than people with low SES [13–17].

One question that arises in this context is whether and to what extent socioeconomic differences in the utilization of medical services are due to socioeconomic differences in healthcare needs [11]. On the one hand, there is evidence from Germany to suggest that the socioeconomic differences in the use of general and specialist medical services can be partially, but not fully, explained by differences in health and morbidity [18]. For example, even in the case of similar health conditions, it was still observed that socially disadvantaged people were more likely than the socially better-off to use GPs or general medical services than specialized medical services [19–21]. On the other hand, other data from Germany indicate that the socioeconomic differences in the use of GPs and specialists disappear almost completely when adjustments are made for people's age and health status [22]. Accordingly, there is a need for further research on this issue.

The present study used nationwide data for Germany to examine the extent to which the utilization of outpatient medical care varies according to a person's SES, and whether socioeconomic differences in outpatient care utilization also exist when the people's states of health are similar. Different medical specializations were examined and gender-specific analyses carried out.

## Material and Methods

### Study design and population

The analyses were based on data from the first wave of the German Health Interview and Examination Survey for Adults (DEGS1), which is part of the national health monitoring system administered by the Robert Koch Institute (RKI) in Berlin. The RKI is a federal institution within the portfolio of the German Federal Ministry of Health responsible for disease control and prevention. DEGS1 was conducted in the period from 2008 to 2011 and included interviews and examinations. The target population was Germany's resident population between the ages of 18 and 79. The sample was drawn from local population registers, supplemented by former participants in the German National Health Interview and Examination Survey 1998 (GNHIES98). A total of 8,152 people took part in DEGS1; 4,193 of these were invited for the first time (response 42%), 3,959 were former participants in the GNHIES98 (response 62%). The study centres were distributed nationwide over 180 sampled communities. The net sample allows for representative cross-sectional analyses of Germany's adult population. The concept, sample design, participants and data-collection methods are described in detail elsewhere [23, 24]. The DEGS1 study protocol was consented with the Federal and State Commissioners for Data Protection and approved by the Charité-Universitätsmedizin Berlin ethics committee in September 2008 (No. EA2/047/08). Participation was voluntary and written informed consent was obtained from all participants prior to the interview and examination. Participants had the possibility to refuse consent to individual aspects of the study, even if they gave overall consent.

### Use of outpatient care

The data on the utilization of outpatient medical services were collected by means of a self-administered questionnaire. Participants were asked to state the number of contacts they had had with office-based physicians over the last twelve months (S1 Appendix). This was followed by a list of different medical specialists; the following specializations were included in the analysis: general medicine, internal medicine, ophthalmology, surgery/orthopaedics, dermatology, gynaecology, otorhinolaryngology (ENT), neurology/psychiatry, psychotherapy (medical/psychological), radiology and urology.

### Socioeconomic status

The SES of the study participants was determined using a multidimensional index developed by the RKI for all surveys conducted within the national health monitoring system in Germany [25]. The index includes information on the educational achievements, occupational status and income of the study participants. The subdimension of educational achievement was covered using the CASMIN educational classification, which takes information on the respondents' school-leaving and vocational qualifications into account [26]. The subdimension of occupational status was determined using the International Socio-Economic Index of Occupational Status (ISEI) according to Ganzeboom et al. [27]. The study participants' income situation was determined via the net equivalent income; for this, household net income was adjusted for household size and age-specific needs of the household members using the modified OECD equivalence scale. This procedure made it possible to take account of the household size and composition in order to determine the study participant's individual financial room for manoeuvre. To calculate the SES index, the three subdimensions—education, occupation, and income—were transferred to three metric subscales with a value range of 1.0 to 7.0. Then the point scores of the three subscales were summed to compute a total score with a value range of 3.0 to 21.0. Finally, the total score was divided into three categories: "low SES" (quintile 1), "middle SES" (quintiles 2–4) and "high SES" (quintile 5) [25].

### Statistical analyses

For the analyses, the study population was limited to people between the ages of 18 and 69, since the accuracy of self-reported data on the utilization of medical services decreases in old age [28] and the proportion of missing values was significantly higher among DEGS1 participants over 70 years of age. The sociodemographic characteristics of the study population are shown in Table 1. The parameters used to indicate the utilization of medical services were the prevalence of utilization (percentage of people with at least one contact with a physician in the last twelve months) and the number of contacts (average number of physician contacts in the last twelve months by people with at least one physician contact). In order to minimize biases due to sampling design and systematic non-response, weighting factors were used in the analysis in order to adjust for sampling probabilities and the sample’s distribution by age, sex, education, nationality, type of municipality and residential region to match the population of Germany on 31 December 2010 [24].

**Table 1:** Sociodemographic characteristics of the study population (n = 6754).

	Men		Women	
	%	*(n)*	%	*(n)*
**Age**				
18–29 years	21.8	*(525)*	21.0	*(547)*
30–39 years	16.9	*(473)*	17.1	*(541)*
40–49 years	24.9	*(716)*	24.2	*(823)*
50–59 years	20.8	*(735)*	21.1	*(857)*
60–69 years	15.6	*(744)*	16.7	*(793)*
**Socioeconomic status**				
Low	18.3	*(480)*	18.4	*(495)*
Middle	58.7	*(1801)*	62.4	*(2213)*
High	23.0	*(883)*	19.2	*(812)*
Missing values	–	*(29)*	–	*(41)*
**Migration background**				
Without/one-sided	84.4	*(2781)*	83.1	*(3099)*
Two-sided	15.6	*(320)*	16.9	*(369)*
Missing values	–	(92)	–	(93)
**Municipality size class**				
Rural	16.2	*(622)*	15.7	*(626)*
Small town	23.1	*(794)*	22.7	*(869)*
Medium-sized town	29.5	*(899)*	30.3	*(1062)*
Major city	31.3	*(878)*	31.4	*(1004)*
**Residential region**				
East (incl. Berlin)	21.1	*(997)*	20.6	*(1120)*
West	78.9	*(2196)*	79.4	*(2441)*

%, weighted percentage (weighted to match the population distribution of Germany on 31 December 2010)

n, unweighted number of cases in the sample

Associations between SES and utilization were analysed using odds ratios (OR) derived from binary logistic regression models. Incidence-rate ratios (IRR) from zero-truncated negative binomial regression models for count data were used to assess associations between SES and the number of physician contacts. In the initial model (Model 1), adjustments were made only for sociodemographic characteristics: age, age^2^, two-sided migration background (yes vs. no) [29], municipality size class (rural, small town, medium-sized town, major city) and residential region (west vs. east including Berlin). In Model 2, subjective and objective indicators of health status were added as control variables: self-rated general health, chronic disease, and global activity limitations—collected using the Minimum European Health Module (MEHM) [30]–as well as self-reported medical diagnoses of coronary heart disease (lifetime prevalence), injury/poisoning, diabetes mellitus, osteoarthritis, rheumatoid arthritis, cancer, depression, anxiety disorder, bronchial asthma, hay fever and atopic eczema (12-month prevalence respectively). The data on self-reported medical diagnoses were collected via computer-assisted personal interviewing (CAPI) [23], while the MEHM was part of the self-administered questionnaire (S1 Appendix). In a further model, additional adjustments were made for the type of health insurance (statutory, private, other) in view of differences between statutory and private health insurance with regard to access to care, the remuneration of medical services, and the composition of policyholders [31, 32]. Finally, the district-level densities of office-based family practitioners, medical specialists and psychotherapists (number per 100,000 inhabitants) were adjusted for to account for potential confounding by regional differences in the availability of outpatient services [33]. The regression models were estimated as random-intercept multilevel models using cluster-robust standard errors, in order to consider the multilevel structure of the data and the clustered sample design. The significance level was set at p < 0.05. All analyses were conducted separately for men and women.

## Results

### General medicine

In total, 75.9% of the men and 81.8% of the women aged between 18 and 69 attended a GP's practice in the last twelve months. The utilization prevalence was higher for men and women with middle SES than for those with high SES (Table 2). After adjusting for sociodemographic characteristics and health indicators, a middle SES remained associated with higher odds of utilization in both sexes. This association no longer existed after additional adjustment for the type of health insurance (S1 Table).

**Table 2:** Utilization of office-based general practitioners by socioeconomic status in men and women.

		Model 1^a^		Model 2^b^	
***Prevalence***	**%**	**OR (95% CI)**	**p-value**	**OR (95% CI)**	**p-value**
**Men**					
Low SES	75.6	1.27 (0.94–1.70)	0.119	1.08 (0.80–1.47)	0.601
Middle SES	77.9	1.34 (1.10–1.64)	0.005	1.26 (1.02–1.56)	0.033
High SES	71.5	1.00		1.00	
**Women**					
Low SES	82.5	1.36 (0.97–1.90)	0.072	1.18 (0.82–1.69)	0.373
Middle SES	83.5	1.38 (1.10–1.74)	0.005	1.31 (1.04–1.67)	0.024
High SES	76.0	1.00		1.00	
***Contacts***	**Ø**	**IRR (95% CI)**	**p-value**	**IRR (95% CI)**	**p-value**
**Men**					
Low SES	5.1	1.53 (1.31–1.79)	0.000	1.25 (1.08–1.46)	0.003
Middle SES	3.7	1.25 (1.15–1.36)	0.000	1.16 (1.08–1.24)	0.000
High SES	2.9	1.00		1.00	
**Women**					
Low SES	4.9	1.49 (1.30–1.71)	0.000	1.20 (1.07–1.34)	0.002
Middle SES	4.0	1.19 (1.08–1.31)	0.001	1.08 (1.00–1.17)	0.038
High SES	3.1	1.00		1.00	

%, 12-month prevalence; OR, odds ratio; Ø, mean number of contacts in the last 12 months; IRR, Incidence rate ratio; CI, confidence interval; SES, socioeconomic status.

^a^ adjusted for age, age^2^, migration background, municipal size class, residential region.

^b^ model 1 plus adjustment for health status (self-rated health, chronic illness, global activity limitations, injury/poisoning, diabetes, coronary heart disease, osteoarthritis, arthritis, cancer, depression, anxiety disorder, asthma, allergic rhinitis, atopic eczema).

Those men and women who used the services of a general practitioner in the last twelve months had on average 3.8 and 4.1 contacts a year, respectively. Patients with low and middle SES had more contacts with general practitioners than those with high SES (Table 2). These differences remained statistically significant after adjusting for sociodemographic characteristics and health indicators. Even after additional adjustment for the type of health insurance and the regional density of outpatient care providers, a low SES remained associated with a significantly higher number of contacts in both sexes (S1 Table).

### Medical specializations

The services of medical specialists in private practice were used at least once in the last twelve months by a total of 64.6% of the men and 89.5% of the women. In Model 1 there were no significant differences by SES among men. However, women with low SES had significantly lower odds of visiting at least one medical specialist than women with high SES (Table 3). After adjusting for sociodemographic characteristics and health indicators (Model 2), the odds of visiting a specialist was significantly lower among both women and men with low SES than among those with high SES. These differences remained even after additional adjustments for the type of health insurance and the regional provider density (S2 Table).

**Table 3:** Utilization of office-based specialists^a^ by socioeconomic status in men and women.

		Model 1^b^		Model 2^c^	
***Prevalence***	**%**	**OR (95% CI)**	**p-value**	**OR (95% CI)**	**p-value**
**Men**					
Low SES	64.9	0.97 (0.74–1.28)	0.844	0.68 (0.51–0.91)	0.009
Middle SES	64.2	1.04 (0.87–1.24)	0.686	0.90 (0.75–1.08)	0.271
High SES	65.0	1.00		1.00	
**Women**					
Low SES	85.1	0.51 (0.33–0.78)	0.002	0.45 (0.30–0.70)	0.000
Middle SES	89.9	0.71 (0.52–0.97)	0.031	0.68 (0.50–0.94)	0.020
High SES	92.8	1.00		1.00	
***Contacts***	**Ø**	**IRR (95% CI)**	**p-value**	**IRR (95% CI)**	**p-value**
**Men**					
Low SES	5.9	1.19 (0.99–1.43)	0.057	0.88 (0.75–1.04)	0.134
Middle SES	4.8	1.07 (0.93–1.23)	0.374	0.92 (0.82–1.04)	0.179
High SES	4.9	1.00		1.00	
**Women**					
Low SES	7.7	1.33 (1.15–1.54)	0.000	1.05 (0.94–1.18)	0.403
Middle SES	5.8	1.04 (0.95–1.14)	0.378	0.96 (0.89–1.04)	0.341
High SES	5.8	1.00		1.00	

%, 12-month prevalence; OR, odds ratio; Ø, mean number of contacts in the last 12 months; IRR, Incidence rate ratio; CI, confidence interval; SES, socioeconomic status.

^a^ specialists in ophthalmology, surgery/orthopaedics, dermatology, gynaecology, otorhinolaryngology, internal medicine, neurology, psychiatry, psychotherapy (also psychological), radiology, urology.

^b^ adjusted for age, age^2^, migration background, municipal size class, residential region.

^c^ model 1 plus adjustment for health status (self-rated health, chronic illness, global activity limitations, injury/poisoning, diabetes, coronary heart disease, osteoarthritis, arthritis, cancer, depression, anxiety disorder, asthma, allergic rhinitis, atopic eczema).

The average number of contacts with specialists was 5.0 among men and 6.3 among women. In this context, no SES differences were found among male patients. By contrast, women patients with low SES had more contacts with specialists on average than those with high SES (Table 3). These differences were also observed after adjustment for sociodemographic characteristics, but disappeared after additionally controlling for indicators of health status (S2 Table).

Table 4 shows the utilization prevalence and odds ratios for at least one visit to an office-based physician over the last twelve months, differentiated according to individual medical specializations. The finding that people with low SES with a similar health status as people with high SES had lower odds of contact with a specialist was particularly clear-cut in both sexes in the fields of internal medicine and dermatology, as well as in gynaecology in the case of women. These differences also remained after controlling for the type of health insurance and regional provider density (S3 Table, S4 Table).

**Table 4:** Utilization of office-based physicians with different medical specialities by socioeconomic status in men and women.

	Men					Women				
		Model 1^a^		Model 2^b^			Model 1^a^		Model 2^b^	
	%	OR (95% CI)	p-value	OR (95% CI)	p-value	%	OR (95% CI)	p-value	OR (95% CI)	p-value
**Ophthalmology**										
Low SES	22.5	1.21 (0.90–1.63)	0.206	1.02 (0.74–1.39)	0.906	29.9	0.85 (0.64–1.12)	0.240	0.76 (0.57–1.01)	0.062
Middle SES	20.5	1.06 (0.87–1.29)	0.580	1.02 (0.83–1.25)	0.863	28.3	0.86 (0.70–1.06)	0.159	0.80 (0.65–1.00)	0.048
High SES	22.1	1.00		1.00		33.2	1.00		1.00	
**Surgery/orthopaedics**										
Low SES	29.2	1.32 (1.01–1.73)	0.042	0.94 (0.70–1.26)	0.669	33.1	1.39 (1.07–1.82)	0.015	1.13 (0.85–1.50)	0.396
Middle SES	30.3	1.43 (1.18–1.73)	0.000	1.21 (0.98–1.50)	0.070	32.2	1.18 (0.98–1.42)	0.076	1.09 (0.90–1.33)	0.364
High SES	24.2	1.00		1.00		26.9	1.00		1.00	
**Dermatology**										
Low SES	16.6	0.64 (0.46–0.89)	0.007	0.61 (0.43–0.84)	0.003	19.9	0.70 (0.54–0.92)	0.010	0.71 (0.53–0.95)	0.020
Middle SES	16.8	0.76 (0.62–0.93)	0.008	0.74 (0.61–0.91)	0.004	21.3	0.76 (0.63–0.93)	0.006	0.76 (0.63–0.93)	0.006
High SES	23.0	1.00		1.00		27.1	1.00		1.00	
**Gynaecology**										
Low SES	–					62.7	0.51 (0.39–0.66)	0.000	0.53 (0.40–0.69)	0.000
Middle SES	–					75.0	0.78 (0.64–0.96)	0.018	0.79 (0.64–0.97)	0.023
High SES	–					79.9	1.00		1.00	
**Otorhinolaryngology**										
Low SES	15.2	0.85 (0.61–1.19)	0.341	0.74 (0.52–1.07)	0.108	16.0	0.83 (0.61–1.14)	0.262	0.75 (0.55–1.02)	0.064
Middle SES	13.6	0.87 (0.69–1.08)	0.197	0.80 (0.64–1.02)	0.067	18.5	0.81 (0.65–1.01)	0.061	0.78 (0.63–0.97)	0.024
High SES	17.7	1.00		1.00		22.0	1.00		1.00	
**Internal medicine**										
Low SES	16.2	0.85 (0.62–1.18)	0.333	0.55 (0.38–0.79)	0.001	15.7	0.74 (0.54–1.02)	0.065	0.53 (0.36–0.76)	0.001
Middle SES	15.5	0.87 (0.68–1.10)	0.240	0.75 (0.58–0.97)	0.032	20.0	0.96 (0.79–1.16)	0.667	0.84 (0.68–1.04)	0.104
High SES	20.4	1.00		1.00		19.5	1.00		1.00	
**Neurology/psychiatry**										
Low SES	8.8	1.31 (0.79–2.16)	0.300	0.80 (0.48–1.35)	0.411	13.3	2.53 (1.69–3.79)	0.000	1.66 (1.06–2.57)	0.025
Middle SES	5.4	1.04 (0.74–1.46)	0.808	0.72 (0.49–1.07)	0.101	8.8	1.37 (1.00–1.88)	0.047	1.11 (0.78–1.58)	0.572
High SES	7.0	1.00		1.00		6.9	1.00		1.00	
**Psychotherapy**^c^										
Low SES	4.4	1.26 (0.67–2.37)	0.481	0.71 (0.32–1.56)	0.392	6.3	1.14 (0.68–1.92)	0.615	0.68 (0.36–1.29)	0.239
Middle SES	2.8	0.85 (0.50–1.44)	0.537	0.52 (0.28–0.95)	0.034	5.6	1.19 (0.79–1.78)	0.409	0.98 (0.63–1.52)	0.924
High SES	3.5	1.00		1.00		5.9	1.00		1.00	
**Radiology**										
Low SES	18.1	1.43 (1.00–2.04)	0.050	0.95 (0.64–1.40)	0.779	19.6	1.11 (0.82–1.51)	0.482	0.89 (0.65–1.22)	0.469
Middle SES	15.0	1.19 (0.95–1.49)	0.139	0.96 (0.75–1.23)	0.738	22.7	1.02 (0.83–1.26)	0.818	0.92 (0.74–1.13)	0.423
High SES	13.1	1.00		1.00		20.7	1.00		1.00	
**Urology**										
Low SES	13.2	0.90 (0.64–1.28)	0.568	0.80 (0.55–1.16)	0.230	6.1	1.88 (1.07–3.32)	0.029	1.55 (0.88–2.74)	0.132
Middle SES	13.6	0.92 (0.72–1.17)	0.485	0.86 (0.66–1.12)	0.253	4.4	1.08 (0.70–1.67)	0.716	0.99 (0.65–1.53)	0.972
High SES	14.7	1.00		1.00		3.4	1.00		1.00	

%, 12-month prevalence; OR, odds ratio; CI, confidence interval; SES, socioeconomic status.

^a^ adjusted for age, age^2^, migration background, municipal size class, residential region.

^b^ model 1 plus adjustment for health status (self-rated health, chronic illness, global activity limitations, injury/poisoning, diabetes, coronary heart disease, osteoarthritis, arthritis, cancer, depression, anxiety disorder, asthma, allergic rhinitis, atopic eczema).

^c^ incl. psychological psychotherapy.

## Discussion

The present study used nationwide data to examine the extent to which the use of outpatient medical services differs between socioeconomic groups. The findings demonstrate that patients with low SES have contact with general practitioners more frequently than those with high SES, even when their health status is similar. By contrast, the use of specialists by people with low SES is lower than among people with high SES when controlling for differences in health and morbidity. This was particularly striking in internal medicine, gynaecology and dermatology. The results, therefore, suggest that socially disadvantaged people are given medical assistance more frequently by GPs than by specialists.

When interpreting the results it should be borne in mind that the data on physician contacts are based on self-reported data. There is a possibility of recall bias in the case of self-reported data, especially if a relatively long recall period like the previous twelve months is covered [28, 34]. However, by comparing survey data with health-insurance data, a validation study conducted in Belgium has shown that there is considerable correspondence between self-reported data and accounting data when it comes to the utilization prevalence [35]. With regard to the number of physician contacts, the self-reported data only slightly underreported utilization. SES characteristics had no independent influence on the correspondence or deviation between self-reported and accounting data.

Further limitations arise from the operationalization of health status, which we included as a proxy for healthcare needs. For a population-based study, the health status was measured relatively comprehensively in the present analysis by considering different subjective and objective indicators of health and disease. Nevertheless, it seems likely that the range and degree of healthcare needs were not completely covered by the health indicators used. In particular, need factors that are specific to certain medical specialist fields could not be considered because the corresponding information was not available in the data. However, this may only have influenced the results if corresponding need factors are also related to SES.

Our findings are similar to results from previous studies. Initial descriptive analyses of the DEGS1 data have already shown that the utilization of GPs is lowest in the high-SES group, while the utilization of specialists in fields such as dermatology and gynaecology are highest in the high-SES group [36]. These findings were confirmed in the present analysis while controlling for health and morbidity and other potential confounders, such as the type of health insurance and regional density of office-based physicians. Also, earlier studies from Germany showed a higher use of GPs by people with low SES, and a higher use of specialized physicians by people with high SES [18, 21, 37]. Similar relationships have been also demonstrated for other European countries [18, 38–40]. A European cross-country comparison showed that social differences in the utilization of specialists exist in many European countries, but that the extent of the differences varies across countries and between different healthcare systems [41]. Higher contact figures for people with low SES can be found on the basis of DEGS1, as well as in other studies [22, 36, 42]. When the focus is on specific physician groups, this is primarily due to more frequent contacts with general practitioners, according to the findings of both this and other studies [37, 43]. When it came to the utilization of medical specialists, the contact figures also hardly differed between people with high and low SES in previous studies [19, 37].

Although our findings show significant SES-related differences in the utilization of GPs and certain medical specialists, it should be borne in mind that such differences in the utilization of medical services do not represent evidence of social inequalities in the quality of healthcare or indicate whether it is in line with people's needs. The question as to whether people with low SES use specialized medical treatment too rarely, or people with high SES do so too frequently, cannot be answered from our findings. Should it be the case that the lower utilization of specialist medical services by people with lower SES is compensated by a higher utilization of GP services, and services of the same quality are substituted within the healthcare fields, then this would not mean a violation of needs-oriented utilization [19]. In future therefore, studies should be conducted not only on the quantity of utilization, but also on the extent to which the quality of healthcare varies with the patients' SES.

Our finding that low SES is associated with more GP visits and a lower utilization of internists, dermatologists, and gynaecologists raises the question of what mechanisms underlie these associations. As this question cannot be answered on the basis of our results, we discuss possible mechanisms and explanations in the following by drawing on the literature. One possible explanation suggested is that SES-related differences in the use of GP and specialist services may be partially due to differences in patient preferences: for example, people with low SES seem to prefer to be treated by a trusted GP than to actively search for a suitable specialist [19, 40]. Conversely, higher SES might be linked with the idea of having a right to be treated by a specialist whenever possible. In this context, people with high SES are likely to benefit from various cognitive and social skills that can be helpful in the search for suitable specialists [44].

Our findings further raise the question of why the association between low SES and lower utilization of specialists can be observed in some medical specialities (internal medicine, dermatology, and gynaecology) and not in others. Here, social differences in preventive orientations and related differences in the uptake of prevention services could play a role. In Germany, cancer screening programmes covered by the statutory health insurance include screenings for cervical cancer, breast cancer, colon cancer, skin cancer, and prostate cancer. Internists, dermatologists, and gynaecologists are involved in the examinations conducted and studies consistently show that people with low SES are less likely than those with high SES to attend such screening examinations [13, 15, 16, 45]. Hence, SES-specific screening participation could have contributed to the observed associations between low SES and lower utilization of internists, dermatologists, and gynaecologists. However, it has to be noted that other specialists, such as urologists, are also involved in cancer screening examinations (e.g. prostate palpation for the early detection of prostate cancer). We did not, however, find any independent associations between SES and the utilization of urologists. Therefore, SES differences in screening participation may not be the only reason for SES differences in the utilization of specialists.

Other possible explanations of the observed SES differences in contacts with medical specialists refer to structural aspects of the healthcare system. For example, socioeconomic differences in the use of specialist services could be caused by access barriers to the healthcare system. In many European countries, people with a low income are more likely to perceive difficulties gaining access to the healthcare system than people with a high income [46]. For Germany there is evidence to suggest that people with a low income forego medical care more frequently than higher-income earners because of financial co-payments, especially since the co-payments constitute a much larger share of their income than for people who are economically better off [47, 48]. This would be particularly relevant in the field of medical specialists if out-of-pocket services or services that require co-payments are offered to patients more often by specialists than by general practitioners. Reference should also be made in this context to the quarterly practice fee that was levied in Germany between 2004 and 2012 (while the data for DEGS1 was being collected). This €10 fee was charged for the first visit to an outpatient physician's practice every quarter and had to be paid by people with statutory health insurance out of their own pocket. Visits to other office-based physicians were then free of charge in the respective quarter if the physician who was first contacted issued a referral. However, if another office-based physician was consulted without a referral, the practice fee had to be paid again. The practice fee is said to have a directing effect on the use of specialists, albeit mainly in the first years after its introduction. For example, people with a low income were more likely than high-income earners forego a visit to the doctor because of the practice fee [49, 50]. However, the observed SES differences in the use of GPs and specialists were also observed in Germany before the introduction of the practice fee [37]. It therefore seems likely that the practice fee is not the primary reason—or perhaps only a partial reason—for the lower use of medical specialists by low-SES groups.

Apart from patient preferences, preventive orientations and potential access barriers, differences in physician-patient communication, as well as in the physicians' referral behaviour, can probably also help explain the socioeconomic differences we observed in the utilization of medical services. An international review [51] reveals that people with low SES communicate less actively when consulting a physician and receive less information from the physician than people with high SES. According to the review, physicians' style of communication towards patients with low SES compared to those with higher SES can be described as more directive, less participatory, less information-giving and less socio-emotional. As a result, physicians often misperceive low-SES patients' desire and need for information as well as their ability to take part in the care process [51, 52]. Interactions between these factors could result in patients with high SES being referred to specialists more quickly than low-SES patients [53, 54]. This in turn may contribute to low-SES people having more GP visits than high-SES people but lower odds of seeing a medical specialist, as we observed in the DEGS1 data for adults in Germany. As this potential explanation could not be established empirically on the basis of the DEGS1 data, future studies should address the influence of patients’ SES on GPs’ referral behaviour and its contribution to SES-related differences in the use of GP and specialist services. Nevertheless, in combination with the previous findings and explanations discussed in the literature, our findings suggest that medical schools and further/advanced training courses for physicians should do more to encourage partnership-based and patient-tailored forms of communication that take the patients' socioeconomic background into account. Wherever possible, this should be done not only in theory, but also by practising practical skills and following role models in hospitals and private practice.

In summary, it can be concluded that SES differences in the use of medical services, which can be observed in several European countries, also exist in the outpatient care sector in Germany. Our findings add to previous work that SES differences in healthcare utilization exist beyond social disparities in health and disease. This indicates that healthcare differs between socially disadvantaged people and those who are socially better-off; however, it should be borne in mind that utilization rates cannot be equated with quality of care. The possible mechanisms underlying the observed utilization differences are diverse and potentially located at different levels; e.g., patients (demand side), physicians (provider side), and the healthcare system. To better understand the factors contributing to SES differences in the use of medical services, future studies should aim at empirically disentangling the respective roles of patient preferences, physician factors, physician-patient interaction, and structural aspects of the healthcare system. Using and combining different methods, such as cross-country comparisons, multi-level analyses, trend analyses (pre and post health system reforms), and qualitative approaches, might be helpful to gain further insights into the underlying mechanism of socioeconomic differences in the utilization of medical care in future research.

## Supporting Information

S1 Appendix Extract of the self-administered questionnaire used in DEGS1. (PDF)

S1 Table Utilization of office-based general practitioners by socioeconomic status in men and women. (PDF)

S2 Table Utilization of office-based specialistsa by socioeconomic status in men and women. (PDF)

S3 Table Utilization of office-based physicians with different medical specialties by socioeconomic status in men. (PDF)

S4 Table Utilization of office-based physicians with different medical specialties by socioeconomic status in women. (PDF)
