# Supplementary material for: Socioeconomic Status and Use of Outpatient Medical Care: The Case of Germany
Source: PLoS One. 2016 May 27;11(5):e0155982. doi: 10.1371/journal.pone.0155982 (PMC4883792; doi:10.1371/journal.pone.0155982)
Supplement: S1 Appendix — (PDF) [file pone.0155982.s001.pdf]

DEGS
Studie zur Gesundheit Erwachsener in Deutschland

ROBERT KOCH INSTITUT

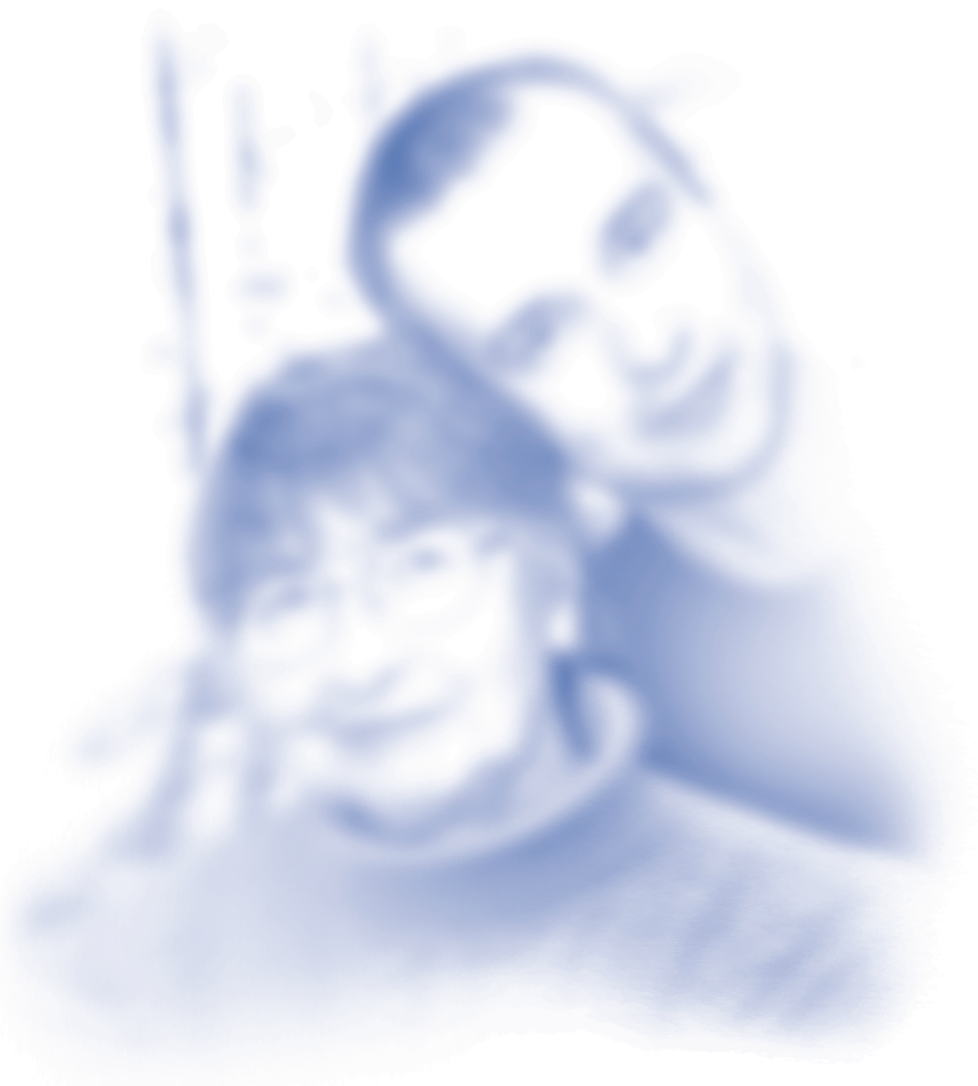

Health Questionnaire

(Exemplary extract)

German Health Interview and Examination Survey for Adults

Personal details

1 **When were you born?**

X	X				1	9		

Day Month Year

2 **What is your sex?**

Male .... ○ Female .... ○

3 **How is your health in general?**

Very good	Good	Fair	Bad	Very bad
○	○	○	○	○

4 **Do you have any long standing chronic illness?**
Chronic illnesses are long-term illnesses necessitating constant treatment and monitoring, e. g. diabetes or heart diseases.

Yes .... ○ No .... ○ Don't know .... ○

5 **For at least the past six months, to what extent have you been limited because of a health problem in activities people usually do?**

Severely limited .... ○
Limited but not severely.. ○
Not limited at all .... ○
Don't know .... ○

9 **Please state how many times in the last 12 months you have seen an office-based doctor practicing in the following fields.**
Please include house calls.

	Yes	Number of times
General practitioner	○	☐☐
Internist (e. g. cardiologist, pneumologist, diabetologist)	○	☐☐
Gynaecologist	○	☐☐
Eye specialist (Ophthalmologist)	○	☐☐
Orthopaedist	○	☐☐
Ear-nose-throat specialist	○	☐☐
Psychiatrist, neurologist	○	☐☐
Medical/psychological psychotherapist	○	☐☐
Surgeon	○	☐☐
Dermatologist	○	☐☐
Radiologist	○	☐☐
Urologist	○	☐☐
Other specialist, please specify (not including therapists): ______	○	☐☐
Dentist, orthodontist	○	☐☐
**I have not seen a doctor in the last 12 months.**	○	

21 **What kind of health insurance cover do you have?**

Statutory health insurance ○

i. e

Allgemeine Ortskrankenkasse (AOK) (statutory health insurance) ○

Ersatzkrankenkasse (e. g. Barmer, TK, DAK, KKH) (alternative health insurance) ○

Betriebskrankenkasse (BKK) (company health insurance) ○

Innungskrankenkasse (IKK) (trade association health insurance) ○

Other insurance provider, e. g. See-Krankenkasse (maritime health insurance), Landwirtschaftliche Krankenkasse (agricultural health insurance), Knappschaft (miners' insurance), Deutsche Rentenversicherung (German pension insurance scheme), Knappschaft-Bahn-See (marine and railway insurance) ○

Do you have a private supplementary insurance policy in addition to your statutory health insurance?

Yes ○ No ○

or

Financial assistance for civil servants ○

Do you have a private supplementary insurance policy in addition to financial assistance for civil servants?

Yes ○ No ○

or

Private health insurance providing full cover ○

or

Foreign health insurance ○

or

Other entitlement to healthcare (e. g. free medical welfare, social-welfare recipient, war-disablement pension, equalization of burdens [Lastenausgleich]) ○

or

Not insured, self-paying patient ○

If you don't know which category your health insurance belongs to, please write the full name of the company you are insured with here:

Injuries and poisonings

22 **Have you had medical treatment for an injury or poisoning in the last 12 months?**

Yes .... ○ No .... ○ → **Please proceed to question 23**

If yes, how many times?

☐☐

122 **Who shares your household with you?**
For example: if you live together with your partner, mother and stepdaughter, enter the information as shown in the following example. Please give their age and sex.

Example:

Person	Age	Sex: Male	Sex: Female
1. Yourself		○	○
2. Partner	38	⊗	○
3. Mother	64	○	⊗
4. Stepdaughter	5	○	⊗

Your details:

Person	Age	Sex: Male	Sex: Female
1. Yourself		○	○
2.		○	○
3.		○	○
4.		○	○
5.		○	○
6.		○	○
7.		○	○
8.		○	○
9.		○	○
10.		○	○

Education

123 What is your highest general school-leaving certificate?

- Elementary school (Haupt-/Volksschule) ○
- Secondary school (Realschule/Mittlere Reife)/ vocational secondary school (Fachschule) ○
- Polytechnic institute qualification ("Polytechnische Oberschule" or POS), or class 10 (before 1965: 8 years) ○
- Technical college qualification (Fachhochschulreife/ Abschluss einer Fachoberschule) ○
- A-levels (Abitur), general or subject-specific qualification for a place at university ○
- Other school-leaving qualification (e. g. obtained abroad) ○
- Left school without qualification ○
- Do not yet have school-leaving qualification ○

124 Are you currently attending a school?

- No ○
- Yes, and I'm aiming for:
  - A lower secondary-school diploma (Hauptschule) ○
  - An intermediate secondary-school diploma (Realschulabschluss/Mittlere Reife/Fachschulreife) ○
  - Technical college qualification (Fachhochschulreife/ Abschluss einer Fachoberschule) ○
  - A-levels (Abitur), general or subject-specific qualification for a place at university ○
  - Other school-leaving qualification ○

125 **What is your <u>highest</u> professional qualification/university degree?**

- Still in professional training (trainee, student, occupational orientation year) ○
- No professional qualification (also did not undergo training) ○
- I have completed a company apprenticeship (Lehre) ○
- I have completed vocational training in college (vocational school [Berufsfachschule], business school, preparation course for the middle grade in public administration [“mittlerer Dienst”]) ○
- I have completed training at specialist college (e. g. master craftsman’s technical college, vocational or specialist training academy) ○
- I have graduated from a college/university with a bachelor’s degree ○
- I have graduated with a different degree from a technical college or an engineering college (Fachhochschule, Ingenieurschule) ○
- I have graduated from a university, polytechnic ○
- Other training qualification ○

126 **Are you currently in vocational training or studying?**

- No ○

Yes, I am doing of the following:

- A company apprenticeship (Lehre) ○
- College training (vocational school [Berufsfachschule], business school, preparation course for the middle grade in public administration [“mittlerer Dienst”]) ○
- Training at technical college (Fachschule, Meisterschule, Technikerschule, Berufsakademie, Fachakademie) ○
- A degree at college/university ○
- A different course of training ○

Career

142 **Have you ever been gainfully employed?**

Yes .... ○ No .... ○ ➔ **Please proceed to question 146**

143 **What is currently your main occupation/job?**
If you are no longer employed, what was your most recent job in your previous main occupation?
Please describe the occupation precisely. Does this occupation/job have a particular name? Please give the exact name, i. e. not "electrician" but "electrical fitter"; not "shop assistant" but "shoe seller"; not "clerk" (Sachbearbeiter) but "accountant" (Buchhalter).

144 **In which sector of the economy are you currently working (or did you last work)?**
Please relate your answer as precisely as possible to the place where you are working/used to work, not to the company as a whole. For example: "machine tool manufacture" (not "factory"), "retail food trade" (not "retail trade"), "hospital" (not "services").

145 **What is your current position at work (or if you are currently unemployed, what was your last position at work)?**

Worker

- Semi-skilled worker ○
- Qualified worker ○
- Qualified worker and specialist ○
- Supervisor, foreman (Vorarbeiter/in, Kolonnenführer/in) ○
- Master craftsman, brigadier (Meister/in, Polier/in, Brigadier/in) ○

Self-employed

Self-employed farmer or member of an agricultural cooperative ("Genossenschaftsbauer") ...

- with up to 10 hectares in agricultural use ○
- with 10 or more hectares in agricultural use ○
- member of an agricultural cooperative ○

Liberal profession (doctor, lawyer, tax consultant, etc.) with ...

- no staff/colleagues ○
- 1–4 staff/colleagues ○
- 5 or more staff/colleagues ○

Other form of self-employment, including "Ich-AG" or "PGH" member, with ...

- no staff/colleagues ○
- 1–4 staff/colleagues ○
- 5 or more staff/colleagues ○
- PGH member (ex-East German cooperative) ○

Public servant, judge, professional soldier ...

- lower grade servant ("einfacher Dienst" up to and including "Oberamtsmeister/in") ○
- clerical service servant ("mittlerer Dienst" from "Assistent/in" up to and including "Hauptsekretär/in", "Amtsinspektor/in)" ○
- higher grade servant ("gehoberner Dienst" from "Inspektor/in" up to and including "Oberamtsrat/-rätin") ○
- Senior civil servant ("höherer Dienst", judge, professor (above "Rat/Rätin") ○

Employee ...

following general instructions (e. g. cashier, shop assistant, typist, janitor/doorman, etc.) ○

qualified job working according to instructions (e. g. clerk, accountant, engineering draughtsman) ○

working independently on responsible tasks or with technical responsibility for staff (e. g. research assistant ("wissenschaftliche/r Mitarbeiter/in"), "Prokurist/in", department manager, employed master craftsman) ○

with comprehensive managerial functions and decision-making authority (e. g. director, general manager, board member) ○

Assisting family member ○

Other (e. g. trainee/apprentice, school pupil, student, person doing military or community service, etc.) ○

146 Are you the main earner in your household?

The main earner is the person with the highest income (including pensions, alimony, social welfare, income from letting and leasing, and other forms of income).

Yes ○ ➔ **Please proceed to question 148**

No ○

147 **What is the main earner's current position at work (or if he/she is currently unemployed, what was his/her most recent position at work)?**

Worker

- Semi-skilled worker ○
- Qualified worker ○
- Qualified worker and specialist ○
- Supervisor, foreman (Vorarbeiter/in, Kolonnenführer/in) ○
- Master craftsman, brigadier (Meister/in, Polier/in, Brigadier/in) ○

Self-employed

Self-employed farmer or member of an agricultural cooperative ("Genossenschaftsbauer") ...

- with up to 10 hectares in agricultural use ○
- with 10 or more hectares in agricultural use ○
- member of an agricultural cooperative ○

Liberal profession (doctor, lawyer, tax consultant, etc.) with ...

- no staff/colleagues ○
- 1–4 staff/colleagues ○
- 5 or more staff/colleagues ○

Other form of self-employment, including "Ich-AG" or "PGH" member, with ...

- no staff/colleagues ○
- 1–4 staff/colleagues ○
- 5 or more staff/colleagues ○
- PGH member (ex-East German cooperative) ○

Public servant, judge, professional soldier ...

- lower grade servant ("einfacher Dienst" up to and including "Oberamtsmeister/in") ○
- clerical service servant ("mittlerer Dienst" from "Assistent/in" up to and including "Hauptsekretär/in", "Amtsinspektor/in)" ○
- higher grade servant ("gehoberner Dienst" from "Inspektor/in" up to and including "Oberamtsrat/-rätin") ○
- Senior civil servant ("höherer Dienst", judge, professor (above "Rat/Rätin") ○

DEGS1

Employee …

following general instructions (e. g. cashier, shop assistant, typist, janitor/doorman, etc.) ○

qualified job working according to instructions (e. g. clerk, accountant, engineering draughtsman) ○

working independently on responsible tasks or with technical responsibility for staff (e. g. research assistant ("wissenschaftliche/r Mitarbeiter/in"), "Prokurist/in", department manager, employed master craftsman) ○

with comprehensive managerial functions and decision-making authority (e. g. director, general manager, board member) ○

Assisting family member ○

Other (e. g. trainee/apprentice, school pupil, student, person doing military or community service, etc.) ○

Income

148 **What is your household's total monthly net income?**
This is the sum of all wages, salaries, income from self-employed work and pensions. Please add any income from public allowances, letting and leasing, housing assistance, child benefits or other sources of income. Then deduct taxes, business expenses and social insurance contributions.

€ ☐ per month ➔ **If you have entered an answer here, please proceed to question 149**

If you can't state the exact amount, please cross one of the categories below:

- Below € 500 ○
- € 500 to below € 750 ○
- € 750 to below € 1,000 ○
- € 1,000 to below € 1,250 ○
- € 1,250 to below € 1,500 ○
- € 1,500 to below € 1,750 ○
- € 1,750 to below € 2,000 ○
- € 2,000 to below € 2,250 ○
- € 2,250 to below € 2,500 ○
- € 2,500 to below € 3,000 ○
- € 3,000 to below € 3,500 ○
- € 3,500 to below € 4,000 ○
- € 4,000 to below € 4,500 ○
- € 4,500 to below € 5,000 ○
- € 5,000 to below € 6,000 ○
- € 6,000 to below € 8,000 ○
- € 8,000 and over ○

Origin

149 **What is your country of birth?**
Please give the name used today, even if the area used to be part of another country (e. g. "Czech Republic", not "Czechoslovakia").

In Germany (in today's borders) .... ○
In another country, ... ○
please specify: [ ]

150 **How long have you been living in the Federal Republic of Germany, i. e. within today's borders (including the former East Germany [GDR])?**

All my life .... ○
Since .... [| | |]

151 **Where were you living in 1988?**

In the former East Germany .... ○
In the former West Germany .... ○
In neither of the above .... ○

152 **Which country was your mother born in?**
Please give the name used today, even if the area used to be part of another country.

In Germany (in today's borders) .... ○
In another country, ... ○
please specify: [ ]

153 **Which country was your father born in?**
Please give the name used today, even if the area used to be part of another country.

In Germany (in today's borders) ○
In another country, ○
please specify: ____

154 **What is/are your nationality/nationalities?**
Please cross all applicable answers.

German ○ **Please proceed to question 156**
Other(s), ○
please specify: ____

155 **Do you have a ...?**

permanent residence permit ○
temporary residence permit ○

156 **What is your native language?**
Please cross all applicable answers.

German ○
Other, ○
please specify: ____

157 **If German isn't your first language, how good would you say your German is?**

Very good	Good	Average	Poor	Bad
○	○	○	○	○

Thank you very much for answering the questions.
