# Supplementary material for: Socioeconomic Status and Use of Outpatient Medical Care: The Case of Germany
Source: PLoS One. 2016 May 27;11(5):e0155982. doi: 10.1371/journal.pone.0155982 (PMC4883792; doi:10.1371/journal.pone.0155982)
Supplement: S1 Table — (PDF) [file pone.0155982.s002.pdf]

**S1 Table. Utilization of office-based general practitioners by socioeconomic status in men and women.**

| | | Model 1[a] | | Model 2[b] | | Model 3[c] | | Model 4[d] | |
|---|---|---|---|---|---|---|---|---|---|
| ***Prevalence*** | **%** | **OR (95% CI)** | **p-value** | **OR (95% CI)** | **p-value** | **OR (95% CI)** | **p-value** | **OR (95% CI)** | **p-value** |
| **Men** | | | | | | | | | |
| Low SES | 75.6 | 1.27 (0.94–1.70) | 0.119 | 1.08 (0.80–1.47) | 0.601 | 0.90 (0.66–1.22) | 0.494 | 0.89 (0.65–1.22) | 0.456 |
| Middle SES | 77.9 | 1.34 (1.10–1.64) | 0.005 | 1.26 (1.02–1.56) | 0.033 | 1.09 (0.87–1.35) | 0.460 | 1.08 (0.89–1.32) | 0.442 |
| High SES | 71.5 | 1.00 | | 1.00 | | 1.00 | | 1.00 | |
| **Women** | | | | | | | | | |
| Low SES | 82.5 | 1.36 (0.97–1.90) | 0.072 | 1.18 (0.82–1.69) | 0.373 | 1.02 (0.71–1.49) | 0.901 | 1.00 (0.72–1.40) | 0.991 |
| Middle SES | 83.5 | 1.38 (1.10–1.74) | 0.005 | 1.31 (1.04–1.67) | 0.024 | 1.16 (0.91–1.49) | 0.228 | 1.15 (0.92–1.44) | 0.217 |
| High SES | 76.0 | 1.00 | | 1.00 | | 1.00 | | 1.00 | |
| ***Contacts*** | **Ø** | **IRR (95% CI)** | **p-value** | **IRR (95% CI)** | **p-value** | **IRR (95% CI)** | **p-value** | **IRR (95% CI)** | **p-value** |
| **Men** | | | | | | | | | |
| Low SES | 5.1 | 1.53 (1.31–1.79) | 0.000 | 1.25 (1.08–1.46) | 0.003 | 1.24 (1.07–1.45) | 0.005 | 1.25 (1.07–1.45) | 0.005 |
| Middle SES | 3.7 | 1.25 (1.15–1.36) | 0.000 | 1.16 (1.08–1.24) | 0.000 | 1.15 (1.07–1.23) | 0.000 | 1.14 (1.06–1.23) | 0.000 |
| High SES | 2.9 | 1.00 | | 1.00 | | 1.00 | | 1.00 | |
| **Women** | | | | | | | | | |
| Low SES | 4.9 | 1.49 (1.30–1.71) | 0.000 | 1.20 (1.07–1.34) | 0.002 | 1.14 (1.02–1.28) | 0.021 | 1.15 (1.01–1.30) | 0.035 |
| Middle SES | 4.0 | 1.19 (1.08–1.31) | 0.001 | 1.08 (1.00–1.17) | 0.038 | 1.04 (0.96–1.13) | 0.279 | 1.05 (0.97–1.13) | 0.270 |
| High SES | 3.1 | 1.00 | | 1.00 | | 1.00 | | 1.00 | |

%, 12-month prevalence; OR, odds ratio; Ø, mean number of contacts in the last 12 months; IRR, incidence rate ratio; CI, confidence interval; SES, socioeconomic status.

[a] adjusted for age, age², migration background, municipality size class, residential region.

[b] model 1 plus adjustment for health status (self-rated health, chronic illness, global activity limitations, injury/poisoning, diabetes, coronary heart disease, osteoarthritis, arthritis, cancer, depression, anxiety disorder, asthma, allergic rhinitis, atopic eczema).

[c] model 2 plus adjustment for type of health insurance (statutory, private, other).

[d] model 3 plus adjustment for the regional density of outpatient care (number of family practitioners, specialists, and psychotherapists per 100,000 inhabitants of the district).
