# Supplementary material for: Socioeconomic Status and Use of Outpatient Medical Care: The Case of Germany
Source: PLoS One. 2016 May 27;11(5):e0155982. doi: 10.1371/journal.pone.0155982 (PMC4883792; doi:10.1371/journal.pone.0155982)
Supplement: S2 Table — (PDF) [file pone.0155982.s003.pdf]

S2 Table. Utilization of office-based specialists[a] by socioeconomic status in men and women.

		Model 1[b]		Model 2[c]		Model 3[d]		Model 4[e]	
Prevalence	**%**	**OR (95% CI)**	**p-value**	**OR (95% CI)**	**p-value**	**OR (95% CI)**	**p-value**	**OR (95% CI)**	**p-value**
**Men**									
Low SES	64.9	0.97 (0.74–1.28)	0.844	0.68 (0.51–0.91)	0.009	0.71 (0.53–0.96)	0.024	0.72 (0.54–0.95)	0.022
Middle SES	64.2	1.04 (0.87–1.24)	0.686	0.90 (0.75–1.08)	0.271	0.93 (0.77–1.13)	0.472	0.93 (0.78–1.12)	0.466
High SES	65.0	1.00		1.00		1.00		1.00	
**Women**									
Low SES	85.1	0.51 (0.33–0.78)	0.002	0.45 (0.30–0.70)	0.000	0.43 (0.28–0.66)	0.000	0.44 (0.29–0.67)	0.000
Middle SES	89.9	0.71 (0.52–0.97)	0.031	0.68 (0.50–0.94)	0.020	0.65 (0.47–0.90)	0.010	0.66 (0.48–0.91)	0.011
High SES	92.8	1.00		1.00		1.00		1.00	
Contacts	**Ø**	**IRR (95% CI)**	**p-value**	**IRR (95% CI)**	**p-value**	**IRR (95% CI)**	**p-value**	**IRR (95% CI)**	**p-value**
**Men**									
Low SES	5.9	1.19 (0.99–1.43)	0.057	0.88 (0.75–1.04)	0.134	0.89 (0.75–1.05)	0.172	0.89 (0.74–1.06)	0.185
Middle SES	4.8	1.07 (0.93–1.23)	0.374	0.92 (0.82–1.04)	0.179	0.92 (0.81–1.05)	0.234	0.93 (0.82–1.05)	0.231
High SES	4.9	1.00		1.00		1.00		1.00	
**Women**									
Low SES	7.7	1.33 (1.15–1.54)	0.000	1.05 (0.94–1.18)	0.403	1.05 (0.93–1.19)	0.435	1.05 (0.93–1.20)	0.422
Middle SES	5.8	1.04 (0.95–1.14)	0.378	0.96 (0.89–1.04)	0.341	0.96 (0.88–1.04)	0.301	0.96 (0.89–1.03)	0.262
High SES	5.8	1.00		1.00		1.00		1.00	

%, 12-month prevalence; OR, odds ratio; Ø, mean number of contacts in the last 12 months; IRR, incidence rate ratio; CI, confidence interval; SES, socioeconomic status.

[a] specialists in ophthalmology, surgery/orthopaedics, dermatology, gynaecology, otorhinolaryngology, internal medicine, neurology, psychiatry, psychotherapy (also psychological), radiology, urology.

[b] adjusted for age, age^2, migration background, municipality size class, residential region.

[c] model 1 plus adjustment for health status (self-rated health, chronic illness, global activity limitations, injury/poisoning, diabetes, coronary heart disease, osteoarthritis, arthritis, cancer, depression, anxiety disorder, asthma, allergic rhinitis, atopic eczema).

[d] model 2 plus adjustment for type of health insurance (statutory, private, other).

[e] model 3 plus adjustment for the regional density of outpatient care (number of family practitioners, specialists, and psychotherapists per 100,000 inhabitants of the district).
