# Supplementary material for: Socioeconomic Status and Use of Outpatient Medical Care: The Case of Germany
Source: PLoS One. 2016 May 27;11(5):e0155982. doi: 10.1371/journal.pone.0155982 (PMC4883792; doi:10.1371/journal.pone.0155982)
Supplement: S3 Table — (PDF) [file pone.0155982.s004.pdf]

S3 Table. Utilization of office-based physicians with different medical specialties by socioeconomic status in men.

		Modell 1[a]		Modell 2[b]		Modell 3[c]		Modell 4[d]	
	%	OR (95% CI)	p-value	OR (95% CI)	p-value	OR (95% CI)	p-value	OR (95% CI)	p-value
**Ophthalmology**									
Low SES	22.5	1.21 (0.90–1.63)	0.206	1.02 (0.74–1.39)	0.906	1.01 (0.72–1.41)	0.946	1.00 (0.72–1.38)	0.978
Middle SES	20.5	1.06 (0.87–1.29)	0.580	1.02 (0.83–1.25)	0.863	1.01 (0.82–1.25)	0.911	1.00 (0.82–1.23)	0.974
High SES	22.1	1.00		1.00		1.00		1.00	
**Surgery/orthopaedics**									
Low SES	29.2	1.32 (1.01–1.73)	0.042	0.94 (0.70–1.26)	0.669	0.95 (0.70–1.29)	0.743	0.94 (0.70–1.27)	0.695
Middle SES	30.3	1.43 (1.18–1.73)	0.000	1.21 (0.98–1.50)	0.070	1.23 (0.99–1.52)	0.061	1.22 (1.00–1.50)	0.055
High SES	24.2	1.00		1.00		1.00		1.00	
**Dermatology**									
Low SES	16.6	0.64 (0.46–0.89)	0.007	0.61 (0.43–0.84)	0.003	0.67 (0.47–0.95)	0.024	0.67 (0.47–0.95)	0.024
Middle SES	16.8	0.76 (0.62–0.93)	0.008	0.74 (0.61–0.91)	0.004	0.80 (0.65–1.00)	0.046	0.80 (0.65–1.00)	0.052
High SES	23.0	1.00		1.00		1.00		1.00	
**Gynaecology**									
Low SES	–								
Middle SES	–								
High SES	–								
**Otorhinolaryngology**									
Low SES	15.2	0.85 (0.61–1.19)	0.341	0.74 (0.52–1.07)	0.108	0.79 (0.54–1.15)	0.218	0.79 (0.56–1.12)	0.190
Middle SES	13.6	0.87 (0.69–1.08)	0.197	0.80 (0.64–1.02)	0.067	0.84 (0.66–1.08)	0.179	0.84 (0.66–1.09)	0.186
High SES	17.7	1.00		1.00		1.00		1.00	
**Internal medicine**									
Low SES	16.2	0.85 (0.62–1.18)	0.333	0.55 (0.38–0.79)	0.001	0.55 (0.37–0.81)	0.002	0.54 (0.36–0.81)	0.003
Middle SES	15.5	0.87 (0.68–1.10)	0.240	0.75 (0.58–0.97)	0.032	0.75 (0.57–1.00)	0.048	0.75 (0.54–1.04)	0.081
High SES	20.4	1.00		1.00		1.00		1.00	

S3 Table *(continued).*

**Neurology/psychiatry**								
Low SES	8.8	1.31 (0.79–2.16)	0.300	0.80 (0.48–1.35)	0.411	0.63 (0.37–1.08)	0.092	0.63 (0.37–1.08)	0.091
Middle SES	5.4	1.04 (0.74–1.46)	0.808	0.72 (0.49–1.07)	0.101	0.59 (0.40–0.88)	0.010	0.58 (0.39–0.87)	0.008
High SES	7.0	1.00		1.00		1.00		1.00	
**Psychotherapy**[e]									
Low SES	4.4	1.26 (0.67–2.37)	0.481	0.71 (0.32–1.56)	0.392	0.79 (0.34–1.84)	0.586	0.79 (0.35–1.77)	0.566
Middle SES	2.8	0.85 (0.50–1.44)	0.537	0.52 (0.28–0.95)	0.034	0.56 (0.29–1.09)	0.090	0.55 (0.28–1.09)	0.087
High SES	3.5	1.00		1.00		1.00		1.00	
**Radiology**									
Low SES	18.1	1.43 (1.00–2.04)	0.050	0.95 (0.64–1.40)	0.779	0.91 (0.61–1.36)	0.650	0.91 (0.63–1.32)	0.627
Middle SES	15.0	1.19 (0.95–1.49)	0.139	0.96 (0.75–1.23)	0.738	0.93 (0.72–1.20)	0.575	0.92 (0.71–1.19)	0.537
High SES	13.1	1.00		1.00		1.00		1.00	
**Urology**									
Low SES	13.2	0.90 (0.64–1.28)	0.568	0.80 (0.55–1.16)	0.230	0.76 (0.51–1.14)	0.185	0.76 (0.51–1.14)	0.183
Middle SES	13.6	0.92 (0.72–1.17)	0.485	0.86 (0.66–1.12)	0.253	0.83 (0.62–1.10)	0.188	0.83 (0.64–1.08)	0.165
High SES	14.7	1.00		1.00		1.00		1.00	

%, 12-month prevalence; OR, odds ratio; CI, confidence interval; SES, socioeconomic status.

[a] adjusted for age, age², migration background, municipality size class, residential region.

[b] model 1 plus adjustment for health status (self-rated health, chronic illness, global activity limitations, injury/poisoning, diabetes, coronary heart disease, osteoarthritis, arthritis, cancer, depression, anxiety disorder, asthma, allergic rhinitis, atopic eczema).

[c] model 2 plus adjustment for type of health insurance (statutory, private, other).

[d] model 3 plus adjustment for the regional density of outpatient care (number of family practitioners, specialists, and psychotherapists per 100,000 inhabitants of the district).

[e] incl. psychological psychotherapy.
