# Supplementary material for: Socioeconomic Status and Use of Outpatient Medical Care: The Case of Germany
Source: PLoS One. 2016 May 27;11(5):e0155982. doi: 10.1371/journal.pone.0155982 (PMC4883792; doi:10.1371/journal.pone.0155982)
Supplement: S4 Table — (PDF) [file pone.0155982.s005.pdf]

S4 Table. Utilization of office-based physicians with different medical specialities by socioeconomic status in women.

		Modell 1[a]		Modell 2[b]		Modell 3[c]		Modell 4[d]	
	%	OR (95% CI)	p-value	OR (95% CI)	p-value	OR (95% CI)	p-value	OR (95% CI)	p-value
**Ophthalmology**									
Low SES	29,9	0,85 (0,64–1,12)	0,240	0,76 (0,57–1,01)	0,062	0,82 (0,60–1,11)	0,203	0,81 (0,61–1,09)	0,169
Middle SES	28,3	0,86 (0,70–1,06)	0,159	0,80 (0,65–1,00)	0,048	0,86 (0,69–1,08)	0,198	0,86 (0,70–1,05)	0,144
High SES	33,2	1,00		1,00		1,00		1,00	
**Surgery/orthopaedics**									
Low SES	33,1	1,39 (1,07–1,82)	0,015	1,13 (0,85–1,50)	0,396	1,16 (0,86–1,56)	0,331	1,16 (0,87–1,55)	0,310
Middle SES	32,2	1,18 (0,98–1,42)	0,076	1,09 (0,90–1,33)	0,364	1,11 (0,91–1,37)	0,308	1,11 (0,93–1,34)	0,239
High SES	26,9	1,00		1,00		1,00		1,00	
**Dermatology**									
Low SES	19,9	0,70 (0,54–0,92)	0,010	0,71 (0,53–0,95)	0,020	0,72 (0,53–0,98)	0,034	0,72 (0,52–0,99)	0,044
Middle SES	21,3	0,76 (0,63–0,93)	0,006	0,76 (0,63–0,93)	0,006	0,77 (0,63–0,94)	0,011	0,77 (0,63–0,93)	0,008
High SES	27,1	1,00		1,00		1,00		1,00	
**Gynaecology**									
Low SES	62,7	0,51 (0,39–0,66)	0,000	0,53 (0,40–0,69)	0,000	0,51 (0,39–0,68)	0,000	0,52 (0,39–0,69)	0,000
Middle SES	75,0	0,78 (0,64–0,96)	0,018	0,79 (0,64–0,97)	0,023	0,77 (0,62–0,95)	0,015	0,77 (0,62–0,97)	0,023
High SES	79,9	1,00		1,00		1,00		1,00	
**Otorhinolaryngology**									
Low SES	16,0	0,83 (0,61–1,14)	0,262	0,75 (0,55–1,02)	0,064	0,78 (0,57–1,09)	0,145	0,79 (0,58–1,09)	0,154
Middle SES	18,5	0,81 (0,65–1,01)	0,061	0,78 (0,63–0,97)	0,024	0,81 (0,64–1,03)	0,080	0,82 (0,65–1,02)	0,078
High SES	22,0	1,00		1,00		1,00		1,00	
**Internal medicine**									
Low SES	15,7	0,74 (0,54–1,02)	0,065	0,53 (0,36–0,76)	0,001	0,51 (0,35–0,75)	0,001	0,52 (0,36–0,75)	0,000
Middle SES	20,0	0,96 (0,79–1,16)	0,667	0,84 (0,68–1,04)	0,104	0,82 (0,66–1,03)	0,082	0,83 (0,67–1,03)	0,085
High SES	19,5	1,00		1,00		1,00		1,00	

S4 Table *(continued).*

**Neurology/psychiatry**									
Low SES	13,3	2,53 (1,69–3,79)	0,000	1,66 (1,06–2,57)	0,025	1,50 (0,96–2,35)	0,077	1,51 (0,96–2,36)	0,075
Middle SES	8,8	1,37 (1,00–1,88)	0,047	1,11 (0,78–1,58)	0,572	1,02 (0,71–1,47)	0,908	1,03 (0,72–1,46)	0,882
High SES	6,9	1,00		1,00		1,00		1,00	
**Psychotherapy[e]**									
Low SES	6,3	1,14 (0,68–1,92)	0,615	0,68 (0,36–1,29)	0,239	0,61 (0,32–1,14)	0,118	0,58 (0,32–1,07)	0,081
Middle SES	5,6	1,19 (0,79–1,78)	0,409	0,98 (0,63–1,52)	0,924	0,88 (0,57–1,37)	0,580	0,87 (0,60–1,26)	0,465
High SES	5,9	1,00		1,00		1,00		1,00	
**Radiology**									
Low SES	19,6	1,11 (0,82–1,51)	0,482	0,89 (0,65–1,22)	0,469	0,92 (0,66–1,28)	0,614	0,94 (0,67–1,31)	0,702
Middle SES	22,7	1,02 (0,83–1,26)	0,818	0,92 (0,74–1,13)	0,423	0,94 (0,74–1,18)	0,584	0,95 (0,76–1,19)	0,661
High SES	20,7	1,00		1,00		1,00		1,00	
**Urology**									
Low SES	6,1	1,88 (1,07–3,32)	0,029	1,55 (0,88–2,74)	0,132	1,40 (0,79–2,45)	0,246	1,40 (0,81–2,42)	0,225
Middle SES	4,4	1,08 (0,70–1,67)	0,716	0,99 (0,65–1,53)	0,972	0,90 (0,59–1,39)	0,648	0,91 (0,60–1,38)	0,646
High SES	3,4	1,00		1,00		1,00		1,00	

%, 12-month prevalence; OR, odds ratio; CI, confidence interval; SES, socioeconomic status.

[a] adjusted for age, age^2, migration background, municipality size class, residential region.

[b] model 1 plus adjustment for health status (self-rated health, chronic illness, global activity limitations, injury/poisoning, diabetes, coronary heart disease, osteoarthritis, arthritis, cancer, depression, anxiety disorder, asthma, allergic rhinitis, atopic eczema).

[c] model 2 plus adjustment for type of health insurance (statutory, private, other).

[d] model 3 plus adjustment for the regional density of outpatient care (number of family practitioners, specialists, and psychotherapists per 100,000 inhabitants of the district).

[e] incl. psychological psychotherapy.
